# Supplementary material for: Universal coverage but unmet need: National and regional estimates of attrition across the diabetes care continuum in Thailand
Source: PLoS One. 2020 Jan 15;15(1):e0226286. doi: 10.1371/journal.pone.0226286 (PMC6961827; doi:10.1371/journal.pone.0226286)
Supplement: S1 Table — SE = standard error. BMI = body mass index. Normoglycemia = fasting plasma glucose < 100 mg/dL and not on treatment. Prediabetes = fasting plasma glucose ≥ 100 mg/dL and not on treatment. BMI categories were: underweight (BMI < 18∙5 kg/m^2), normal (18∙5 ≤ BMI < 25), overweight (25 ≤ BMI < 30), and obese (BMI ≤ 30). Estimates for overall population and by sex, BMI, religion, educational level, geography, and region were age-standardized using five-year categories between 20–70+ using the 2010 Thai Census population estimates. (DOCX) [file pone.0226286.s003.docx]

**Supplementary Table 1: Prevalence of normoglycemia, prediabetes, and diabetes in Thailand, 2014**

|  | **Normoglycemia** | | | | **Prediabetes** | | | **Diabetes** | | | |
| --- | --- | --- | --- | --- | --- | --- | --- | --- | --- | --- | --- |
|  | **Percent** | **SE** | | | **Percent** | | **SE** | **Percent** | | **SE** | |
| Age Standardized | 74∙9 | 0∙55 | | | 16∙3 | | 0∙49 | 8∙82 | | 0∙31 | |
| Crude | 70∙9 | 0∙53 | | | 18∙0 | | 0∙44 | 11∙1 | | 0∙34 | |
| **Age Categories (years)** |  |  | | |  | |  |  | |  | |
| 20-29 | 87∙3 | 1∙25 | | | 9∙80 | | 1∙12 | 2∙86 | | 0∙6 | |
| 30-39 | 79∙9 | 1∙31 | | | 15∙4 | | 1∙21 | 4∙71 | | 0∙61 | |
| 40-49 | 73∙1 | 1∙09 | | | 17∙8 | | 0∙93 | 9∙13 | | 0∙74 | |
| 50-59 | 62∙7 | 1∙12 | | | 21∙8 | | 0∙95 | 15∙5 | | 0∙83 | |
| 60-69 | 56∙4 | 1∙1 | | | 22∙8 | | 0∙93 | 20∙8 | | 0∙89 | |
| 70+ | 59∙0 | 1∙4 | | | 22∙1 | | 1∙16 | 18∙9 | | 1∙12 | |
| **Sex** |  |  | | |  | |  |  | |  | |
| Female | 75∙9 | 0∙74 | | | 14∙9 | | 0∙65 | 9∙26 | | 0∙43 | |
| Male | 73∙9 | 0∙83 | | | 17∙9 | | 0∙74 | 8∙28 | | 0∙45 | |
| **Sex by Age** |  |  | | |  | |  |  | |  | |
| F20-29 | 86∙9 | 1∙77 | | | 10∙4 | | 1∙64 | 2∙73 | | 0∙77 | |
| F30-39 | 82∙3 | 1∙67 | | | 12∙6 | | 1∙51 | 5∙14 | | 0∙86 | |
| F40-49 | 74∙6 | 1∙46 | | | 16∙2 | | 1∙19 | 9∙13 | | 1∙05 | |
| F50-59 | 64∙4 | 1∙46 | | | 20∙0 | | 1∙22 | 15∙6 | | 1∙1 | |
| F60-69 | 54∙7 | 1∙48 | | | 21∙7 | | 1∙24 | 23∙6 | | 1∙25 | |
| F>=70 | 58∙9 | 1∙9 | | | 20∙3 | | 1∙51 | 20∙8 | | 1∙58 | |
| M20-29 | 87∙8 | 1∙75 | | | 9∙20 | | 1∙52 | 2∙99 | | 0∙93 | |
| M30-39 | 77∙3 | 2∙06 | | | 18∙5 | | 1∙94 | 4∙22 | | 0∙85 | |
| M40-49 | 71∙3 | 1∙64 | | | 19∙5 | | 1∙43 | 9∙12 | | 1∙02 | |
| M50-59 | 60∙9 | 1∙71 | | | 23∙6 | | 1∙47 | 15∙5 | | 1∙26 | |
| M60-69 | 58∙2 | 1∙64 | | | 24∙1 | | 1∙41 | 17∙6 | | 1∙26 | |
| M>=70 | 59∙3 | 2∙04 | | | 24∙5 | | 1∙79 | 16∙2 | | 1∙51 | |
| **BMI** |  |  | | |  | |  |  | |  | |
| Underweight | 80∙0 | 1∙78 | | | 13∙3 | | 1∙45 | 6∙69 | | 1∙15 | |
| Normal | 75∙2 | 0∙69 | | | 15∙9 | | 0∙58 | 8∙91 | | 0∙44 | |
| Overweight | 64∙4 | 1 | | | 21∙1 | | 0∙84 | 14∙5 | | 0∙68 | |
| Obese | 60∙9 | 1∙79 | | | 23∙4 | | 1∙56 | 15∙6 | | 1∙15 | |
| **Religion** |  |  | | |  | |  |  | |  | |
| Buddhist | 74∙4 | 0∙59 | | | 16∙6 | | 0∙52 | 8∙96 | | 0∙33 | |
| Not Buddhist | 79∙9 | 1∙53 | | | 13∙2 | | 1∙35 | 6∙85 | | 0∙89 | |
| **Highest Educational Level** | | |  |  | |  | | |  | |  |
| Primary or less | 73∙7 | 1∙31 | | | 16∙3 | | 1∙12 | 10∙0 | | 0∙77 | |
| Low secondary | 71∙9 | 1∙49 | | | 18∙7 | | 1∙32 | 9∙38 | | 0∙96 | |
| High secondary or vocational | 76∙4 | 1∙1 | | | 16∙1 | | 0∙98 | 7∙47 | | 0∙63 | |
| University | 77∙4 | 1∙5 | | | 15∙6 | | 1∙32 | 7∙03 | | 0∙92 | |
| **Geography** |  |  | | |  | |  |  | |  | |
| Rural | 74∙0 | 0∙79 | | | 17∙0 | | 0∙7 | 9∙06 | | 0∙47 | |
| Urban | 75∙7 | 0∙76 | | | 15∙6 | | 0∙69 | 8∙63 | | 0∙4 | |
| **Region** |  |  | | |  | |  |  | |  | |
| Bangkok | 78∙8 | 1∙25 | | | 13∙1 | | 1∙1 | 8∙07 | | 0∙73 | |
| South | 77∙8 | 1∙13 | | | 16∙1 | | 1∙06 | 6∙11 | | 0∙5 | |
| North | 77∙3 | 1∙14 | | | 15∙2 | | 1∙02 | 7∙52 | | 0∙63 | |
| Central | 69∙8 | 1∙17 | | | 19∙3 | | 1∙03 | 10∙8 | | 0∙71 | |
| Northeast | 74∙3 | 1∙17 | | | 16∙1 | | 1∙05 | 9∙53 | | 0∙66 | |
