# Supplementary material for: Universal coverage but unmet need: National and regional estimates of attrition across the diabetes care continuum in Thailand
Source: PLoS One. 2020 Jan 15;15(1):e0226286. doi: 10.1371/journal.pone.0226286 (PMC6961827; doi:10.1371/journal.pone.0226286)
Supplement: S2 Table — SE = standard error. BMI = body mass index. BMI categories were: underweight (BMI < 18∙5 kg/m^2), normal (18∙5 ≤ BMI < 25), overweight (25 ≤ BMI < 30), and obese (BMI ≤ 30). Estimates for overall population and by sex, BMI, religion, educational level, geography, and region were age-standardized using five-year categories between 20–70+ using the 2010 Thai Census population estimates. Source: NHES-V (DOCX) [file pone.0226286.s004.docx]

**Supplementary Table 2: Prevalence of unscreened, undiagnosed, untreated, and uncontrolled diabetes, among total Thai population 2014**

|  | **Unscreened** | | | | **Undiagnosed** | | **Untreated** | | **Uncontrolled** | | **Controlled** | |
| --- | --- | --- | --- | --- | --- | --- | --- | --- | --- | --- | --- | --- |
|  | **Percent** | | **SE** | | **Percent** | **SE** | **Percent** | **SE** | **Percent** | **SE** | **Percent** | **SE** |
| Age Standardized | 1∙64 | | 0∙2 | | 2∙74 | 0∙19 | 0∙09 | 0∙03 | 0∙85 | 0∙08 | 3∙5 | 0∙15 |
| Crude | 1∙5 | | 0∙16 | | 3∙28 | 0∙2 | 0∙13 | 0∙05 | 1∙18 | 0∙11 | 5∙02 | 0∙21 |
| **Age Categories (years)** |  | |  | |  |  |  |  |  |  |  |  |
| 20-29 | 1∙87 | | 0∙52 | | 0∙85 | 0∙31 | 0 | 0 | 0 | 0 | 0∙14 | 0∙07 |
| 30-39 | 1∙62 | | 0∙4 | | 1∙99 | 0∙39 | 0∙01 | 0∙01 | 0∙28 | 0∙12 | 0∙82 | 0∙23 |
| 40-49 | 1∙91 | | 0∙39 | | 3∙25 | 0∙46 | 0∙09 | 0∙07 | 1∙1 | 0∙29 | 2∙78 | 0∙37 |
| 50-59 | 1∙41 | | 0∙3 | | 4∙63 | 0∙5 | 0∙33 | 0∙18 | 2∙1 | 0∙33 | 7∙07 | 0∙55 |
| 60-69 | 0∙83 | | 0∙18 | | 5∙18 | 0∙5 | 0∙13 | 0∙07 | 2∙25 | 0∙28 | 12∙41 | 0∙73 |
| 70+ | 0∙7 | | 0∙19 | | 4∙12 | 0∙57 | 0∙11 | 0∙08 | 1∙42 | 0∙28 | 12∙51 | 0∙97 |
| **Sex** |  | |  | |  |  |  |  |  |  |  |  |
| Female | 1∙14 | | 0∙23 | | 2∙99 | 0∙29 | 0∙08 | 0∙04 | 1∙1 | 0∙14 | 3∙95 | 0∙21 |
| Male | 2∙17 | | 0∙32 | | 2∙47 | 0∙24 | 0∙09 | 0∙05 | 0∙58 | 0∙08 | 2∙97 | 0∙21 |
| **Sex by Age** |  | |  | |  |  |  |  |  |  |  |  |
| F20-29 | 1∙33 | | 0∙58 | | 1∙21 | 0∙5 | 0 | 0 | 0 | 0 | 0∙19 | 0∙12 |
| F30-39 | 1∙01 | | 0∙43 | | 2∙69 | 0∙63 | 0 | 0 | 0∙43 | 0∙21 | 1 | 0∙35 |
| F40-49 | 1∙47 | | 0∙53 | | 3∙71 | 0∙69 | 0∙17 | 0∙14 | 1∙48 | 0∙51 | 2∙31 | 0∙42 |
| F50-59 | 1∙08 | | 0∙38 | | 3∙76 | 0∙55 | 0∙13 | 0∙09 | 2∙77 | 0∙52 | 7∙82 | 0∙8 |
| F60-69 | 0∙43 | | 0∙13 | | 5∙18 | 0∙7 | 0∙14 | 0∙11 | 2∙72 | 0∙44 | 15∙12 | 1∙05 |
| F>=70 | 0∙4 | | 0∙18 | | 3∙82 | 0∙68 | 0∙13 | 0∙13 | 1∙54 | 0∙39 | 14∙9 | 1∙44 |
| M20-29 | 2∙39 | | 0∙85 | | 0∙51 | 0∙36 | 0 | 0 | 0 | 0 | 0∙09 | 0∙09 |
| M30-39 | 2∙31 | | 0∙69 | | 1∙18 | 0∙4 | 0∙02 | 0∙02 | 0∙09 | 0∙06 | 0∙61 | 0∙29 |
| M40-49 | 2∙39 | | 0∙56 | | 2∙76 | 0∙59 | 0 | 0 | 0∙68 | 0∙24 | 3∙29 | 0∙63 |
| M50-59 | 1∙74 | | 0∙46 | | 5∙55 | 0∙85 | 0∙53 | 0∙35 | 1∙39 | 0∙39 | 6∙28 | 0∙76 |
| M60-69 | 1∙29 | | 0∙36 | | 5∙18 | 0∙73 | 0∙13 | 0∙09 | 1∙71 | 0∙33 | 9∙32 | 1 |
| M>=70 | 1∙11 | | 0∙37 | | 4∙52 | 0∙97 | 0∙08 | 0∙08 | 1∙26 | 0∙37 | 9∙27 | 1∙14 |
| **BMI** |  | |  | |  |  |  |  |  |  |  |  |
| Underweight | 1∙33 | | 0∙47 | | 1∙46 | 0∙43 | 0 | 0 | 0∙91 | 0∙52 | 1∙09 | 0∙33 |
| Normal | 1∙72 | | 0∙28 | | 2∙35 | 0∙26 | 0∙06 | 0∙03 | 0∙64 | 0∙11 | 2∙45 | 0∙16 |
| Overweight | 1∙14 | | 0∙32 | | 3∙36 | 0∙38 | 0∙15 | 0∙08 | 1∙1 | 0∙16 | 4∙81 | 0∙3 |
| Obese | 2∙39 | | 0∙63 | | 3∙73 | 0∙52 | 0∙14 | 0∙09 | 1∙21 | 0∙28 | 6∙79 | 0∙7 |
| **Religion** |  | |  | |  |  |  |  |  |  |  |  |
| Buddhist | 1∙66 | | 0∙21 | | 2∙86 | 0∙2 | 0∙09 | 0∙03 | 0∙86 | 0∙09 | 3∙5 | 0∙15 |
| Not Buddhist | 1∙56 | | 0∙58 | | 0∙96 | 0∙28 | 0 | 0 | 0∙8 | 0∙24 | 3∙52 | 0∙58 |
| **Highest Educational Level** |  |  | |  | | |  |  |  |  |  |  |
| Primary or less | 2∙91 | | 0∙7 | | 2∙55 | 0∙25 | 0∙07 | 0∙03 | 0∙92 | 0∙12 | 3∙58 | 0∙21 |
| Low secondary | 1∙73 | | 0∙46 | | 3∙75 | 0∙69 | 0∙19 | 0∙14 | 0∙62 | 0∙22 | 3∙1 | 0∙5 |
| High secondary or vocational | 0∙97 | | 0∙25 | | 2∙3 | 0∙36 | 0∙09 | 0∙06 | 0∙69 | 0∙2 | 3∙42 | 0∙44 |
| University | 1∙33 | | 0∙45 | | 2∙47 | 0∙59 | 0∙04 | 0∙02 | 0∙58 | 0∙2 | 2∙6 | 0∙55 |
| **Geography** |  | |  | |  |  |  |  |  |  |  |  |
| Rural | 1∙83 | | 0∙31 | | 2∙83 | 0∙28 | 0∙03 | 0∙01 | 0∙86 | 0∙13 | 3∙52 | 0∙21 |
| Urban | 1∙45 | | 0∙23 | | 2∙68 | 0∙25 | 0∙16 | 0∙07 | 0∙86 | 0∙09 | 3∙49 | 0∙21 |
| **Region** |  | |  | |  |  |  |  |  |  |  |  |
| North | 1∙23 | | 0∙38 | | 2∙28 | 0∙4 | 0∙02 | 0∙02 | 0∙67 | 0∙15 | 3∙32 | 0∙3 |
| Central | 2∙56 | | 0∙49 | | 3∙34 | 0∙43 | 0 | 0 | 0∙79 | 0∙14 | 4∙14 | 0∙29 |
| Northeast | 1∙83 | | 0∙43 | | 3∙18 | 0∙38 | 0∙03 | 0∙02 | 1∙2 | 0∙22 | 3∙29 | 0∙3 |
| South | 0∙69 | | 0∙23 | | 1∙51 | 0∙24 | 0∙09 | 0∙05 | 0∙68 | 0∙13 | 3∙15 | 0∙37 |
