# Supplementary material for: Universal coverage but unmet need: National and regional estimates of attrition across the diabetes care continuum in Thailand
Source: PLoS One. 2020 Jan 15;15(1):e0226286. doi: 10.1371/journal.pone.0226286 (PMC6961827; doi:10.1371/journal.pone.0226286)
Supplement: S3 Table — Nested logistic regression. Multivariable adjusted odds ratios estimated using continuation ratio logit model with coefficients freely varying across stages. Analysis incorporated sample weights. aOR = adjusted odds ratio. BMI = body mass index. BMI categories were: underweight (BMI < 18∙5 kg/m^2), normal (18∙5 ≤ BMI < 25), overweight (25 ≤ BMI < 30), and obese (BMI ≤ 30). For health system variables (hospitalization per population, staff per population, health center per population, public health nurses per population), values were standardized so a one unit increase represents a one standard deviation increase from the mean. Source: NHES-V. (DOCX) [file pone.0226286.s005.docx]

**Supplementary Table 3: Factors associated with diabetes care cascade retention, Thailand 2014. Nested logistic regression.**

|  | **Screened** | | | | | **Diagnosed** | | | | | | **Controlled** | | | | | |
| --- | --- | --- | --- | --- | --- | --- | --- | --- | --- | --- | --- | --- | --- | --- | --- | --- | --- |
|  | **aOR** | **95% CI** | | | **p** | **aOR** | **95% CI** | | | | **p** | **aOR** | **95% CI** | | | | **p** |
| **Region** |  | |  |  |  |  | |  |  |  | |  | |  |  |  | |
| Northeast | 1 | |  |  |  | 1 | |  |  |  | | 1 | |  |  |  | |
| Bangkok + Central | 0∙60 | | 0∙30 | 1∙20 | 0∙15 | 0∙96 | | 0∙62 | 1∙49 | 0∙86 | | 1∙99 | | 1∙05 | 3∙74 | 0∙03 | |
| South | 0∙83 | | 0∙36 | 1∙93 | 0∙67 | 1∙37 | | 0∙82 | 2∙30 | 0∙23 | | 1∙58 | | 0∙80 | 3∙12 | 0∙19 | |
| North | 0∙74 | | 0∙38 | 1∙44 | 0∙37 | 1∙08 | | 0∙71 | 1∙65 | 0∙71 | | 2∙15 | | 1∙09 | 4∙24 | 0∙03 | |
| **Age** |  | |  |  |  |  | |  |  |  | |  | |  |  |  | |
| Age in 10 year increments | 2∙62 | | 2∙12 | 3∙25 | <0∙001 | 1∙50 | | 1∙30 | 1∙72 | <0∙001 | | 1∙59 | | 1∙32 | 1∙91 | <0∙001 | |
| **Sex** |  | |  |  |  |  | |  |  |  | |  | |  |  |  | |
| Female | 1 | |  |  |  | 1 | |  |  |  | | 1 | |  |  |  | |
| Male | 0∙38 | | 0∙23 | 0∙61 | <0∙001 | 0∙78 | | 0∙59 | 1∙05 | 0∙103 | | 1∙37 | | 0∙89 | 2∙10 | 0∙15 | |
| **BMI** |  | |  |  |  |  | |  |  |  | |  | |  |  |  | |
| Underweight | 0∙46 | | 0∙17 | 1∙21 | 0∙12 | 0∙71 | | 0∙31 | 1∙65 | 0∙43 | | 0∙74 | | 0∙19 | 2∙77 | 0∙65 | |
| Normal | 1 | |  |  |  | 1 | |  |  |  | | 1 | |  |  |  | |
| Overweight | 2∙35 | | 1∙39 | 3∙98 | 0∙001 | 1∙36 | | 0∙99 | 1∙87 | 0∙06 | | 1∙16 | | 0∙75 | 1∙80 | 0∙51 | |
| Obese | 1∙28 | | 0∙68 | 2∙43 | 0∙44 | 1∙69 | | 1∙12 | 2∙56 | 0∙01 | | 1∙69 | | 0∙91 | 3∙12 | 0∙095 | |
| **Highest Educational Level** |  | |  |  |  |  | |  |  |  | |  | |  |  |  | |
| Primary or Lower | 1 | |  |  |  | 1 | |  |  |  | | 1 | |  |  |  | |
| Low Secondary | 1∙48 | | 0∙67 | 3∙29 | 0∙33 | 0∙64 | | 0∙38 | 1∙09 | 0∙10 | | 1∙48 | | 0∙63 | 3∙48 | 0∙37 | |
| High Secondary or Vocational | 1∙95 | | 0∙94 | 4∙05 | 0∙07 | 0∙84 | | 0∙52 | 1∙38 | 0∙49 | | 1∙42 | | 0∙65 | 3∙06 | <0∙001 | |
| University | 1∙03 | | 0∙42 | 2∙54 | 0∙94 | 0∙77 | | 0∙40 | 1∙50 | 0∙45 | | 1∙35 | | 0∙50 | 3∙64 | 0∙55 | |
| **Geography** |  | |  |  |  |  | |  |  |  | |  | |  |  |  | |
| Rural | 1 | |  |  |  | 1 | |  |  |  | | 1 | |  |  |  | |
| Urban | 0∙92 | | 0∙56 | 1∙51 | 0∙74 | 0∙9 | | 0∙66 | 1∙22 | 0∙51 | | 0∙84 | | 0∙56 | 1∙26 | 0∙401 | |
| **Health System** |  | |  |  |  |  | |  |  |  | |  | |  |  |  | |
| Hospital per Population, standardized | 0∙63 | | 0∙39 | 1∙01 | 0∙06 | 0∙80 | | 0∙60 | 1∙07 | 0∙14 | | 1∙04 | | 0∙70 | 1∙55 | 0∙84 | |
| Health Center per Population, Standardized | 2∙33 | | 1∙24 | 4∙39 | 0∙01 | 1∙39 | | 0∙97 | 2∙00 | 0∙08 | | 1∙23 | | 0∙71 | 2∙14 | 0∙46 | |
| Staff per Population, standardized | 2∙49 | | 1∙03 | 6∙01 | 0∙04 | 2∙17 | | 1∙24 | 3∙81 | 0∙01 | | 0∙80 | | 0∙37 | 1∙75 | 0∙58 | |
| Public Health Nurses per Population, Standardized | 0∙94 | | 0∙53 | 1∙67 | 0∙84 | 0∙68 | | 0∙46 | 0∙99 | 0∙05 | | 1∙08 | | 0∙64 | 1∙80 | 0∙78 | |
| Subpopulation (n) | 2255 | | | | | 2064 | | | | | | 1480 | | | | | |
