# Supplementary material for: Universal coverage but unmet need: National and regional estimates of attrition across the diabetes care continuum in Thailand
Source: PLoS One. 2020 Jan 15;15(1):e0226286. doi: 10.1371/journal.pone.0226286 (PMC6961827; doi:10.1371/journal.pone.0226286)
Supplement: S4 Table — Brant test of parallel regression assumption tests whether coefficients for a specific independent variable is statistically different across sequential ordinal outcomes, in this case screened, diagnosed, or controlled diabetes. The null hypothesis is that all coefficients are the same. P values > 0∙05 indicate the null hypothesis is true, ie coefficients are the same across outcomes. P values <0∙05 indicate evidence to reject the null hypothesis, ie coefficients are different across outcomes. (DOCX) [file pone.0226286.s006.docx]

**Supplementary Table 4: Brant test on independent variable coefficients across outcomes of screened, diagnosed, and controlled**

|  | **Chi2** | **p>Chi2** |
| --- | --- | --- |
| All | 75∙2 | <0∙001 |
| **Region** |  |  |
| North | 1 |  |
| Bangkok + Central | 0∙31 | 0∙86 |
| South | 2∙69 | 0∙26 |
| Northeast | 13∙6 | 0∙001 |
| **Age** |  |  |
| Age in 10 year increments | 17∙3 | <0∙001 |
| **Sex** |  |  |
| Female | 1 |  |
| Male | 13∙1 | 0∙001 |
| **BMI** |  |  |
| Underweight | 1 |  |
| Normal | 7∙31 | 0∙03 |
| Overweight | 9∙85 | 0∙01 |
| Obese | 5∙07 | 0∙08 |
| **Highest Educational Level** |  |  |
| Primary or Lower | 1 |  |
| Low Secondary | 3∙98 | 0∙14 |
| High Secondary or Vocational | 1∙30 | 0∙52 |
| University | 0∙62 | 0∙73 |
| **Geography** |  |  |
| Rural | 1 |  |
| Urban | 1∙66 | 0∙44 |
| **Health System** |  |  |
| Hospital per Population, standardized | 6∙45 | 0∙04 |
| Staff per Population, standardized | 13∙7 | 0∙001 |
| Health Center per Population, Standardized | 5∙22 | 0∙07 |
| Public Health Nurses per Population, Standardized | 3∙60 | 0∙17 |
